# Spirituality and mood pathology in severe skin conditions: a prospective observational study

**DOI:** 10.1007/s00403-016-1672-5

**Published:** 2016-07-04

**Authors:** Human-Friedrich Unterrainer, M. Lukanz, M. Pilch, S. Scharf, M. Glawischnig-Goschnik, N. Wutte, R. Fink-Puches, E. Aberer

**Affiliations:** 1 Department of Psychiatry, Medical University of Graz, Graz, Austria; 2 Department of Dermatology and Venereology, Medical University of Graz, Graz, Austria; 3 Department of Medical Psychology and Psychotherapy, Medical University of Graz, Graz, Austria; 4 Center for Integrative Addiction Research (CIAR), Grüner Kreis Society, Rudolfsplatz 9, 1090 Vienna, Austria

**Keywords:** Mood disorders, Lupus erythematosus, Melanoma, Spiritual well-being, Systemic sclerosis

## Abstract

Although the association between spirituality and parameters of psychological health and disease has been investigated extensively, little evidence is available for its potential role in dermatology. In a single-centre observational prospective study, 149 outpatients (107 women) with systemic sclerosis (SSc; *n* = 44), lupus erythematosus (LE; *n* = 48), or early stage malignant melanoma (MM; *n* = 57) were investigated using the multidimensional inventory for religious/spiritual well-being together with the Brief Symptom Inventory for psychiatric symptoms (BSI-18). SSc patients reported the highest amount of Somatization in comparison with LE and MM patients (*p* < 0.05). Furthermore, in line with the previous research, spiritual dimensions, such as Hope for a better future (*p* < 0.01) or Hope for a better afterlife (*p* < 0.01), proved to be especially negatively predictive for the global amount of psychiatric symptom burden in these dermatological patient groups. Our findings suggest that greater attention should be given to spiritual issues, such as encouraging patients, imbuing them with optimism, and offering interventions that address spiritual well-being.

## Introduction

In recent years, there has been a growing interest in finding the link between religious/spiritual issues and improved quality of life in severe illness and their expected salutogenic effect on well-being in this setting [15]. Accordingly, it has been suggested that the bio-psycho-social model of health and disease might be fruitfully augmented by considering a religious/spiritual component in handling patients [11]. This notion, however, has also been considered speculative or its role exaggerated [17]. In any case, the influence of the spiritual dimension in skin disease, as being related to different parameters of mental health, is still poorly investigated, although this area of research seems to have great potential [8]. Religion as being related to various parameters of mental health and illness was prominently described as “the search for significance in ways related to the sacred” (p. 11) [13]. Consequently, spirituality, as one of the key elements of religion, was overwhelmingly confirmed to be negatively related with mood pathology [12]. Mood disorders as being detrimental to quality of life, in turn, were reported in approximately 30 % of dermatology patients [1, 7]. More recently, it was reported in a large multicentre study performed in 13 European countries that of the 3635 dermatological patients who participated in the study, depression was present in 10.1 %, anxiety in 17.2 %, and suicidal ideation in 12.7 % of the cases [5].

Given this data, the purpose of the present study was to do pioneering work in addressing the anti-depressive potential of spirituality with regard to dermatologic patients. We were particularly interested in finding out which of the various dimensions of religion and spirituality, as examined by the multidimensional inventory for religious/spiritual well-being, were related to mood pathology and psychiatric symptoms.

## Materials and methods

### Study design and participants

With the expertise of authors specialising in dermatology, we conducted a prospective single-centre observational study with consecutive patients from the outpatient clinic for autoimmune disease, oncology, and the day clinic at the department of dermatology and venereology, Medical University of Graz, between March and October 2013. Ethics approval was obtained from the ethics committee of the Medical University of Graz, Austria (25-280 ex12/13). Patients with systemic sclerosis (SSc) and lupus erythematosus (LE) (both chronic and potentially life-threatening diseases), and those with early stages of malignant melanoma (MM) without metastasis but with an unknown disease course were administered questionnaires by two members (ML and SS) of our research group and were asked to complete them after written consent was obtained immediately after their doctor’s appointment. The participants received no remuneration or reward for participation. SSc was classified according to Le Roy’s criteria [10] and LE according to the Duesseldorfer classification and the American College of Rheumatology criteria (ACR) [9, 20]. MM patients had stage I–II melanoma diagnosed by histologic criteria and sentinel node biopsy.

### Psychometric measures

The Austrian-German standardized and normed multidimensional inventory of religious/spiritual well-being (MI-RSWB) [23] measures spiritual well-being which is a subjective phenomenon, consisting in equal measures of existential well-being (EWB) for the immanent area of perception, such as Hope immanent (HI), forgiveness (FO) and experiences of sense and meaning (SM), and religious well-being (RWB) for the transcendent area, such as general religiosity (GR), connectedness (CO), and hope transcendent (HT). In addition, marker items are given as examples to illustrate the meaning of the different dimensions: general religiosity: “My faith gives me a feeling of security”; connectedness: “I have experienced the feeling of being absorbed into something greater”; forgiveness: “There are things which I cannot forgive” (coded reversely); experiences of sense and meaning: “I have experienced true (authentic) feelings”; hope immanent: “I view the future with optimism”; hope transcendent: “I often think about the fact that I will have to leave behind my loved ones” (coded reversely). The total amount of all six sub-scales, Religious/Spiritual Well-Being (RSWB), has been defined as “the ability to experience and integrate meaning and purpose in existence through a connectedness with self, others, or a power greater than oneself” (p. 117) [21]. Each sub-scale consists of six items (thus a total score of 48 items) and has to be answered on a six-point Likert scale ranging from 1 to 6. In the previous research, Cronbach’s *α* was found to be at least 0.68 for all the sub-scales and 0.89 for the RSWB total score [23]. A full item list for the English version of the scale together with a short manual can be retrieved from Unterrainer et al. [22].

The Brief Symptom Inventory-18 (BSI-18) is a short version of the highly established Symptom Checklist SCL-90-R [6]. The amount of psychiatric burden in three dimensions of psychiatric symptoms (somatization, depression, and anxiety) for the preceding 7 days is assessed by means of 18 items (six items for each sub-scale). The BSI-18 includes a five-point rating form ranging from 1 (absolutely not) to 5 (very strong). It is also possible to collate the 18 items into a total score: the Global Severity Index (GSI) of psychiatric symptoms. In the previous research, Cronbach’s *α* was observed to be at least 0.79 for all the sub-dimensions and 0.91 for total Global Severity Index score (GSI) [6].

### Statistical methods

Univariate and multivariate general linear models were conducted to investigate differences in mood pathology between the different dermatological groups. The correlation between the RSWB dimensions and mood pathology was tested by Pearson’s correlation statistics. For in-depth analysis, linear regression modelling was used for those RSWB dimensions predicting the global psychiatric symptom burden (GSI). Due to the exploratory nature of the study, the alpha level of significance was set to 0.05.

## Results

242 patients were recruited; 182 gave their signed consent. 33 patients were excluded due to various reasons (e.g., they did not return the questionnaire or did not complete it). Thus, the data of 149 patients (107 women) with SSc (*n* = 44; 37 women; age range 23–80), LE (*n* = 48; 41 women; age range 24–78), and MM (*n* = 57; 29 women; age range 23–80) were available for the analysis. Patients were between 23 and 80 years of age (M = 53.50, SD = 14.02). 107 (72 %) patients were in a relationship or married; 42 (28 %) were single, separated, widowed, or divorced; 114 (76.5 %) had children; 33 (22.1 %) patients had a higher education; and 122 (81.9 %) patients were affiliated with a religious community. These affiliations are as follows: 112 (75.2 %) were Roman Catholic, 8 (5.4 %) were Protestant, 1 (0.7 %) was affiliated with an alternate Christian sect, 1 (0.7 %) stated another (non-Christian) religion and 2 (1.3 %) declined to answer the question.

The subtypes of SSc were limited (59 %), diffuse (16 %), undifferentiated (14 %), and primary Raynaud’s phenomenon (11 %). Of LE patients, 71 % had cutaneous LE and 29 % had systemic LE. Sentinel node biopsy was performed on 62 % of MM patients, and no metastases were detected. More detailed patient data were published in a recent paper [14].

As shown in Table 1, we did not observe any differences in global psychiatric symptom burden as determined by the BSI-18 among the patient groups; however, SSc patients exhibited a higher amount of Somatization compared to MM patients (*p* < 0.05). Compared to a group of inpatients undergoing psychotherapy [6], our group of skin disease patients showed, in general, a substantially lower amount of mood pathology (*p* < 0.001).

**Table 1:** Differences in mood pathology in different skin disease groups

	SSc		LE	MM	*F*	*p*	*η* ^2^	Post hoc
	Alpha	M (SD)	M (SD)	M (SD)				
Somatization	0.79	5.18 (3.99)	4.81 (4.35)	2.77 (3.64)	3.35	<0.05	0.04	SSc > MM
Anxiety	0.83	4.59 (4.57)	4.85 (4.5)	3.42 (4.34)	0.66	n.s.		
Depression	0.89	3.68 (5.02)	3.56 (4.09)	2.32 (4.18)	1.18	n.s.		
Global Severity Index	0.92	13.43 (11.92)	13.21 (10.89)	8.51 (10.91)	1.92	n.s.		

MANCOVA (*df*_error_ = 141; *p* < 0.05) controlled for age and gender

*SSc* systemic sclerosis patients (*n* = 44), *LE* lupus erythematosus patients (*n* = 48), *MM* malignant melanoma patients (*n* = 57)

As revealed by Table 2, hope for a better future (HI; *p* < 0.01) was observed to be the strongest negative predictor of global psychiatric symptom burden (GSI). General religiosity (GR; *p* < 0.01) and hope transcendent (HT; *p* < 0.01) showed a minor but still significant impact on patients’ condition. Notably, there was a positive correlation between the Connectedness dimension and mood pathology (*p* < 0.01). Overall, the assumption of a negative correlation between RSWB and the GSI was confirmed; however, this is mainly due to the contribution of more existentially oriented dimensions of well-being (EWB; *p* < 0.01), while more religiously oriented parameters (RWB) did not show any significant effect (*p* > 0.05).

**Table 2:** Relationship (correlation and regression coefficients) between RSWB dimensions and parameters of mood pathology in dermatologic patients

	Somatization		Anxiety		Depression		Global Severity Index	
	*r*	*β*	*r*	*β*	*r*	*β*	*r*	*β*
SM	0.16	0.27**	0.03	0.09	0.02	0.07	0.08	0.16
HI	−0.24**	−0.40**	−0.33**	−0.38**	−0.40**	−0.49**	−0.37**	−0.48**
FO	−0.15	0.01	−0.21*	−0.04	−0.20*	0.01	−0.22**	−0.01
HT	−0.23**	−0.14	−0.31**	−0.27**	−0.36**	−0.29**	−0.34**	−0.27**
GR	−0.04	−0.20*	−0.11	−0.27**	−0.04	−0.24**	−0.07	−0.27**
CO	0.14	0.22*	0.12	0.28**	0.20*	0.39**	0.18*	0.34**
Corr. *R* ^2^		0.17		0.24		0.35		0.33
RSWB	−0.10		−0.23**		−0.22**		−0.21*	
EWB	−0.12	−0.11	−0.25**	−0.23**	−0.29**	−0.30**	−0.25**	−0.25**
RWB	−0.05	−0.01	−0.14	−0.06	−0.07	0.04	−0.10	−0.01
Corr. *R* ^2^		0.00		0.05		0.07		0.05

Pearson correlation and beta coefficients of the linear regression model

*SM* experiences of sense and meaning, *HI* hope immanent, *FO* forgiveness, *HT* hope transcendent, *GR* general religiosity, *CO* connectedness, *RSWB* religious/spiritual well-being total score, *EWB* existential well-being, *RWB* religious well-being

* *p* < 0.05; ** *p* < 0.01

## Discussion

In this study, we focused on the role of spirituality and its function in the mood stabilization of different patient groups with severe skin diseases. Our main finding was that all parameters of mood pathology were negatively associated with existential and, to a minor extent, with religious well-being. Most significantly, hope immanent (for a better future) was confirmed as being the strongest negative correlate of mood pathology. More unexpectedly, hope transcendent (for a better afterlife) also turned out as a distinct predictor of more adequate adjustment to a chronic/life-threatening skin disease. In line with the previous findings [1], we observed an increased amount of somatization in SSc compared to MM patients. This could also be the cause of reduced physical well-being as published recently [24]. Interestingly, we found that the dimension of connectedness (feeling connected with the universe) was positively related to all parameters of psychiatric symptom burden. This is in contrast to recent research which mostly demonstrated the salutogenic effect of the feeling of “being connected” in anxious/depressive inpatients, assessed by means of different parameters, such as sense of coherence or more adequate coping strategies. However, in conjunction with the previous work, we assumed that feeling connected to the universe might mirror feelings of alienation and isolation from the real world in this specific patient group [23]. In more recent studies, we could show that patients with a skin disease exhibited a substantially lower level of experiences of sense and meaning (*p* < 0.001) in comparison with the general population. Patients with LE had a lower religious/spiritual well-being (*p* < 0.001), while patients with LE and MM had a lower level of general religiosity (*p* < 0.001) and religious well-being (*p* < 0.001). By contrast, MM patients exhibited a higher level of forgiveness (*p* < 0.01) and hope transcendent (*p* < 0.01) and a lower level of connectedness (*p* < 0.001). No differences were found for hope immanent and the amount of existential well-being in skin disease patients compared to the general population [24].

In line with the previous research [23], we conclude that our initial results, concerning different groups of patients with severe skin conditions, confirm the hypothesis that there exists a high therapeutic potential of the spiritual dimension in clinical treatment. However, these results still have to be confirmed by employing larger samples incorporating centres in other countries and different dermatologic patient groups, such as metastasizing melanoma, psoriasis, or atopic dermatitis. Further studies might also consider the fact that other variables, such as personality traits, could mediate or moderate the correlations in addition to poor QoL that might have also influenced the outcome of the current study in SSc and LE patients [3]. Therefore, more research is needed to make a more general statement about the role of spirituality in skin diseases. The dimension of hope, especially, seems to be of central relevance for the mood stability of patients dealing with these severe skin diseases. Further research might focus now on the development of spiritually integrated therapeutic interventions, such as supplying patients, with hope or discussing existential questions as a potential treatment for dermatological patients [2]. In fact, there is already a lot of literature on how to integrate spiritual dimensions, such as hope or sense, of meaning most effectively into patient treatment [19]. Hope therapy [18] was reported to increase some psychological strengths and reduce some symptoms of psychopathology [4]. Furthermore, the core-dimension of existential therapy [16] of finding a meaning in life for psychological well-being can be found prominently discussed in the literature [25].
